# Parametric studies on droplet generation reproducibility for applications with biological relevant fluids

**DOI:** 10.1002/elsc.201700086

**Published:** 2017-09-27

**Authors:** Stefan Wiedemeier, Marko Eichler, Robert Römer, Andreas Grodrian, Karen Lemke, Krees Nagel, Claus‐Peter Klages, Gunter Gastrock

**Affiliations:** ^1^ Bioprocess Engineering Institute for Bioprocessing and Analytical Measurement Techniques e.V. (iba) HeilbadHeiligenstadt Germany; ^2^ Atmospheric Pressure Processes Fraunhofer Institute for Surface Engineering and Thin Films (IST) Braunschweig Germany

**Keywords:** Droplet based microfluidics, Droplet characterization, Droplet media, Droplet reproducibility, Surface coating

## Abstract

Although the great potential of droplet based microfluidic technologies for routine applications in industry and academia has been successfully demonstrated over the past years, its inherent potential is not fully exploited till now. Especially regarding to the droplet generation reproducibility and stability, two pivotally important parameters for successful applications, there is still a need for improvement. This is even more considerable when droplets are created to investigate tissue fragments or cell cultures (e.g. suspended cells or 3D cell cultures) over days or even weeks. In this study we present microfluidic chips composed of a plasma coated polymer, which allow surfactants‐free, highly reproducible and stable droplet generation from fluids like cell culture media. We demonstrate how different microfluidic designs and different flow rates (and flow rate ratios) affect the reproducibility of the droplet generation process and display the applicability for a wide variety of bio(techno)logically relevant media.

**Abbreviations**
1D: One dimensional
2D: Two dimensional
3D: Three dimensional
AFM: Atomic force microscopy
c – C_4_F_8_: ring‐shaped perfluorocyclobutane
DBD: Dielectric barrier discharge
DMEM: Dulbecco's Modified Eagle's Medium
DMS: Droplet generation micro‐system
PC: Polycarbonat
PFD: Perfluorodecaline
SP: Syringe pump

## 1 Introduction

The tremendous progress of droplet based microfluidic technologies over the past few years reveals its high potential for applications in both academia and industry 1, 2, 3, 4. Due to the extremely low achievable droplet volume microfluidic technologies can easily be used to either optimize or even replace state‐of‐the‐art technologies employed in high throughput applications. The promising developments of microfluidic technologies open up new perspectives in the fields of life sciences, chemistry and pharmaceutical research 5, 6, 7, 8, 9, 10, 11.

**Figure 1:**
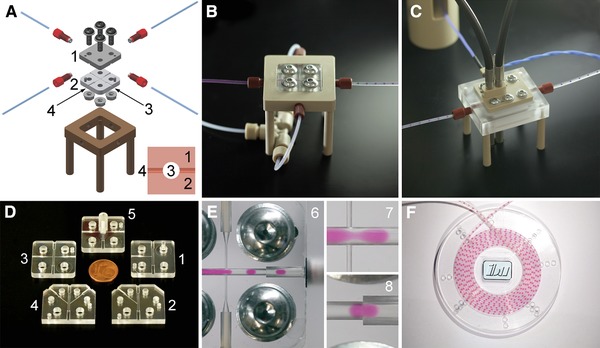
The experimental setup is composed of fluidic modules. (A)‐DMS including mounting frame (1: top plate, 2: bottom plate, 3: main channel, 4: side channel) and (B)‐Photo of (A). (C)‐Detection module with two optical detectors (OD). (D)‐Five different DMS designs 1‐5. (E)‐Image 6: Droplet generation (2D flow‐focusing) from cell culture medium (red). Images 7 and 8: details from image 6. (F)‐Droplet storage disk.

The droplet based microfluidic approach is an aliquoting one based on the immiscibility of normally two fluids (Fig. 1E). The resulting individual segments (droplets) have water like properties and can be considered as non‐touching reaction vessels with a volume ranging from pico‐ to microliters. Compared to typical high‐throughput reaction vessels like microtiter plates (MTP) with a typical volume of 10 μL per well for a 1536 MTP, the droplet volumes are significantly smaller and additionally protected from evaporation and contamination because they can be stored in micro channels or tubes. In addition to just creating droplets microfluidic devices are also capable of manipulating/processing droplets, i.e. substances can be added to the droplets and droplets can be mixed, stored, cultivated and analyzed 12, 13, 14. These processes were already successfully demonstrated for suspensions of prokaryotes, eukaryotes and even multi‐cellular assemblies in tiny volumes 15, 16, 17, 18.

This broad range of biological applications is associated with the use of highly diverse culture media possessing different properties regarding e.g. viscosity and surface energy. As culture medium components like proteins or salts significantly determine the surface energy it is obvious that the droplet generation process as well as the droplet behavior inside the micro channels highly depends on the concentration of these components. In this study we concentrate on the characterization of the droplet generation process depending on the kind of aqueous (culture) medium. The investigations focus on the droplet generation micro‐system (DMS) which is of pivotal importance for a reproducible droplet generation process and thus for future reliable assay results. Two key parameters of the DMS have to be tailored in order to achieve a high quality droplet generation: (i) provide perfect geometrical conditions for the reproducible generation of droplets from the aqueous phase and (ii) reduce the adhesion of the aqueous phase to the channel walls past the droplet generation zone.

In the first part of this study the reproducibility of the droplet generation from cell culture medium as the disperse (aqueous) phase as a function of the DMS design and the applied flow rates was assessed 19, 20, 21, 22, 23. The second part of the study additionally addresses the applicability of the DMS to generate droplets from a broad spectrum of bio(techno)logically relevant and challenging media.

## 2 Materials and methods

### 2.1 Droplet generation micro‐system (DMS)

Each DMS was assembled from two polycarbonate (PC) plates (24 mm x 24 mm x 4 mm, Fig. 1A) which were home made by precision manufacturing (milling, drilling) using a commercial PC plate with 4 mm thickness. The microfluidic channels were milled using a radius cutter resulting in half rounded channels in each PC plate (Fig. 1D). Additionally, two pin bores as well as four bores for screws to compress the PC plates were precisely drilled into each PC plate. Using two pins the two PC plates could be adjusted during assembling them to the complete DMS which features circular micro channels (main channel: 1 mm diameter, side channel: 0.3 mm diameter, Fig. 1A). To compress the PC plates four screws and nuts including washers were used. The torque for each screw fixing was 2 Nm which guarantees liquid tightness for PC plates with thicknesses >3 mm and exact surface flatness.

To avoid the adhesion of the aqueous phase to the micro channel walls the milled surfaces of the PC plates were plasma coated. As alternative to plasma coating, surfactants could be used which normally serve to stabilize the droplet sequence 24. However, surfactants could cause a transfer of matter between droplets separated by oil 25. For this reason the use of surfactants was excluded for the experiments described in this study. After coating the PC plates were assembled face to face by screw based joining facilitated by adjusting pins and drillings (reversible process, Fig. 1A) before they were mounted into a frame that served for tubing port (Fig. 1A).

In order to investigate droplet generation processes and their reproducibility five different DMS designs were manufactured and tested (Table 1 and Fig. 1D). For all DMS designs the main channel was used to guide the aqueous phase to the junction. The functionality was tuned by either injecting perfluorodecaline (PFD, continuous fluidic phase) through one side channel or through two side channels or through an annular slit. For the 3D DMS (Fig. 1D, design 5) a cone element was implemented to create an annular gap with a width of 150 μm which is arranged in an angle of 45° around the main channel (3D flow‐focusing) allowing for a 3D injection of PFD during the droplet generation process.

**Table 1:** DMS designs used for the experiments

DMS No.	Description	Dimension	Name	Reference	DMS Figure
1	One 90° side channel	1D (junction)	1D 90° DMS	Fig. 1(D), No. 1	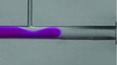
2	One 45° side channel	1D (junction)	1D 45° DMS	Fig. 1(D), No. 2	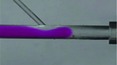
3	Two 90° side channels	2D (flow‐focusing)	2D 90° DMS	Fig. 1(D), No. 3	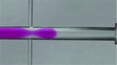
4	Two 45° side channels	2D (flow‐focusing)	2D 45° DMS	Fig. 1(D), No. 4	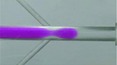
5	45° annular slit	3D (flow‐focusing)	3D DMS	Fig. 1(D), No. 5	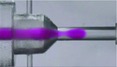

The experiments were performed employing a modular experimental setup which included a syringe pump system (neMESYS, cetoni GmbH, Germany), the droplet generation micro‐system DMS (Fig. 1A and 1B), the home made optical detection micro‐system OMS (Fig. 1C) and a home made storage disk (Fig. 1F). Polytetrafluoroethylene (PTFE) tubes were used to guide the fluids and the droplets. For the determination of the droplet length and the droplet velocity the voltage signals of two optical detectors (OD), connected via fiber optics with the OMS (Fig. 1C), were analyzed. The voltage signals depended on a change of the optical absorption at 525 nm when the droplets passed through. From these signals both the droplet length and droplet flow velocity could be calculated.

However, since during the experiments deviations from idealized droplet shapes could be observed, a correction factor T_s/s_ had to be introduced. This factor represents the ratio of the droplet length average s_D,SP_, calculated from the sample volume delivered by the syringes pump (SP) and divided by the number of generated droplets n_max_, and the droplet length average s_D,OD_ determined by the optical detection (Eq. (1)):

(1) $$T_{s/s}=\frac{s_{D,SP}}{s_{D,OD}}$$

In Eq. (2) the correction factor T_s/s_ was used to calculate the length s_TD,nOD_ of the non‐deformed droplets:

(2) $$s_{TD,nOD}=s_{D,nOD}\;\cdot T_{s/s}$$

From this corrected s_TD,nOD_ the exact droplet volumes were calculated. Subsequently, the volume of each individual droplet was determined employing this correction factor that is specific for each experiment. All processes including droplet generation, droplet detection and all calculations were performed using a home made C++‐procedure.

### 2.2 Coating process

Atmospheric‐pressure plasma‐activated chemical vapor deposition (PA‐CVD) of perfluorinated amorphous carbon films, then termed a‐C:F in analogy with hydrogenated “diamond‐like” carbon films, a‐C:H, was achieved using a “corona”‐activated CVD process with tetrafluoroethylene C_2_F_4_ as precursor 26. The term “corona” was at that time applied in according to industrial customs but the used plasma was in fact not a corona discharge in the strict physical sense but a dielectric barrier discharge (DBD), stabilized by an insulator. Appreciable deposition rates between 100 and 200 nm/min were achieved and surface free energies as low as 16 mN/m were measured on the relatively soft, smooth coatings, using contact angle measurements with six different liquids. Details about DBD‐activated PA‐CVD growth of perfluorinated hydrophobic plasma polymers were reported by Vinogradov and Lunk 27. The techniques used for the deposition of the films used in the present study are described in more recent papers 28. As precursor perfluorocyclobutane (c‐C_4_F_8_, Linde, Pullach, Germany, 4.8) was used as a mixture with argon (5.0) containing 5% (v/v) c‐C_4_F_8_. The discharge was powered by a TIGRES V20‐901.

### 2.3 Physico‐chemical investigations

#### 2.3.1 Surface energy

The surface free energy (including polar and dispersive components) was determined employing the OWRK approach (Owens, Wendt, Rabel und Kaelble). Briefly, contact angles for the four fluids deionized water, formamide, ethyleneglycol (predominantly polar) and diiodomethane (disperse) were recorded using the OCA System (dataphysics GmbH, Germany, sessil drop, 3 droplet of 3 μL each). The investigations were performed on milled polycarbonate plates with a roughness of S_a_ =  0 .79 μm +/− 0.05 μm before and after the plasma coating procedure.

#### 2.3.2 Film thickness via atomic force microscopy (AFM)

Part of the plasma film was removed from a plasma coated glass slide with a scalpel. The thickness of the film was determined at this artificially created edge employing the NanoWizard AFM (JPK Instruments AG, Germany, scan area: 15 × 15 μm^2^, resolution: 512 × 512 pixels).

### 2.4 Influence of DMS designs and flow rates

Experiments with five DMS designs and various flow rate sets were performed to investigate their influence on both, the droplet volume and the droplet volume reproducibility. All deviations were evaluated using the coefficient of variation (CV). The experiments were performed using Dulbecco's Modified Eagle's Medium (DMEM, Sigma‐Aldrich Chemie GmbH, Germany, product number D5523, supplemented with 4.5 g/L *D*‐glucose, 2 mmol/L *L*‐glutamine, 100 U/mL penicillin, 100 μg/mL streptomycin, 10% (v/v) fetal calf serum and 0.01% (w/v) phenol red). All experiments were performed with PFD as continuous hydrophobic phase.

Prior to the experiment the DMS was intensively rinsed with PFD (all channels and tubing). The experiments were performed with PFD flow rates Q_c_ of 250 μL/min, 500 μL/min and 1000 μL/min and a respective ratio of Q_c_ to Q_d_ (flow rate of DMEM as disperse phase) of 10, 5 and 2.5 (Table 2).

**Table 2:** Regime of the droplet generation experiments

	Series 1			Series 2		
Flow rate ratio Qc/Qd	Experiment number	First run	Second run	Third run	4th run	Experiment no.
		Flow rate combination Qc/Qd	Flow rate combination Qc/Qd	Flow rate combination Qc/Qd	Flow rate combination Qc /Qd	
10/1	1	250/25		250/25		13
5/1	2–3	250/50	250/50	250/50	250/50	14–15
2.5/1	4	250/100		250/100		16
10/1	5	500/50		500/50		17
5/1	6–7	500/100	500/100	500/100	500/100	18–19
2.5/1	8	500/200		500/200		20
10/1	9	1000/100		1000/100		21
5/1	10–11	1000/200	1000/200	1000/200	1000/200	22–23
2.5/1	12	1000/400		1000/400		24
	Deviation	Intra‐run	Intra‐run	intra‐run	intra‐run	
		Inter‐run		Inter‐run		
		Total inter‐run				

To investigate the droplet generation reproducibility in terms of the flow rate, various experiments with nine different flow rate combinations were performed. These nine experiments representing the first run started with the flow rate combination 250/25 and ended up with the flow rate combination 1000/400 (Table 2). To evaluate the short term behavior of the DMS, the experiments with the flow rate ratio of 5/1 were performed twice representing the second run. Consequently, twelve experiments were performed with the same DMS.

To investigate the long term behavior of the DMS the first run and the second run experiments were repeated as third and 4th run using the same DMS. The delay between Series 1 and Series 2 was ∼48 h. To evaluate the droplet generation reproducibility three deviation types were defined (Table 2):

- intra‐run‐deviation (standard deviation for each experiment of a single run),
- inter‐run‐deviation (standard deviation only for the 5/1 ratio experiments of the first and the second run as well as for the third and the 4th run),
- total inter‐run‐deviation (standard deviation for Series 1 and Series 2 experiments with the same flow rate combination).

### 2.5 Investigations on the droplet generation of biomedical and biotechnologically relevant samples

In order to estimate the applicability of the coating for bio(techno)logically and biomedically relevant applications, various challenging aqueous media were used for droplet generation employing the 2D 90° DMS (flow rate sets: 250/25, 500/50, 500/100, 1000/100 and 1000/200). These media comprise anticoagulated blood products (whole blood, plasma and serum), Iscove's modified dulbecco's medium (IMDM, see Table 3) 29 and DMEM, a cell suspension (yeast cells) and media with a high protein and fat content. Furthermore, droplet generation was performed using media with varying pH values (pH 2 to pH 10), high glucose concentration, high salinity, high viscosity (alginate) and the not‐fluorinated tetradecane. For order numbers and providers of the used media and suspensions see Table 3. All experiments were performed with PFD as continuous hydrophobic phase.

**Table 3:** Quality of the droplet generation process for application relevant media (including their composition)

Sample media	Specifications	Process stability
Whole blood	Porcine blood + 0.37% (w/v) Sodium citrate dihydrate	0
Human plasma	Blood plasma; ITM Suhl gGmbH (Germany)	0
FBS	Product No.: S 0115 FBS (calf) Biochrom GmbH (Germany)	+
DMEM	see "Materials and methods"	+
IMDM	Iscove's modified Dulbecco's Medium Product No.: 31980030 Thermo Scientific GmbH (Germany); supplemented with 20% (v/v) FBS, 2 mmol/L L‐Glutamine, 1% (v/v) nonessential aminoacids and 100 μmol/L β‐mercaptoethanol	+
Yeast suspension	3 * 107 cfu/mL (Rhodotorula mucilaginosa) in YPD‐Medium (10 g/L Yeastextract; 20 g/L Peptone; 20 g/L *D*‐Glucose; pH 6.5)	+
PBS	8 g/L NaCl; 0.24 g/L KH2PO4; 0.2 g/L KCl; 1.8 g/l Na2HPO4×2 H2O; pH 7.4	+
pH 2 solution	PBS adjusted to pH 2 with HCl	−
pH 4 solution	PBS adjusted to pH 4 with HCl	+
pH 8 solution	PBS adjusted to pH 8 with NaOH	+
pH 10 solution	PBS adjusted to pH 10 with NaOH	−
Glucose solution	PBS + 90.1 g/L *D*‐Glucose	+
Saline solution	PBS + 146.1 g/L NaCl	+
Alginate solution	5 g/L Alginate + 0.9 g/L NaCl Product No. (Alginate): W201502 Sigma‐Aldrich Chemie GmbH (Germany)	+
Concentrated milk	7.5% fat content; Tchibo GmbH (Germany)	+
Double cream	20% fat content; Milbona (Lidl Stiftung & Co. KG; Germany)	+
Tetradecane	Product No.: 87140 Sigma‐Aldrich Chemie GmbH (Germany)	+

For this proof of concept experiment series, the reproducibility of the process was evaluated qualitatively. The scale for this qualitative evaluation ranges from “ + ”: droplet generation without any adhesion; via “ 0 ”: slightly adhesion of the developing droplet with the channel wall to “ − ”: heavily disturbed droplet generation process with significant adhesion.

When media interfered with the DMS channel surface, the DMS was subsequently rinsed with deionized water and PFD. Finally, DMEM was used as disperse phase to investigate whether the observed effect was reversible.

## 3 Results and discussion

### 3.1 Characterization of the surface coating

The uncoated PC surface displayed a water contact angle of 88.3° ± 1.4° and a surface tension of 40.4 mN/m (39.6 mN/m dispersive and 0.7 mN/m polar components). After plasma coating with the precursor c‐C_4_F_8_ the water contact angle increased to 120.0° ± 3°). Furthermore, the surface energy decreased to 10.6 mN/m (the dispersive and polar components decreased to 10.4 mN/m and 0.2 mN/m, respectively). The coated surfaces also displayed oleophobic properties (limited wettability with the not‐fluorinated tetradecane) but they highly supported the adhesion of fluorinated oils like PFD (PFD contact angle of 0°).

**Figure 2:**
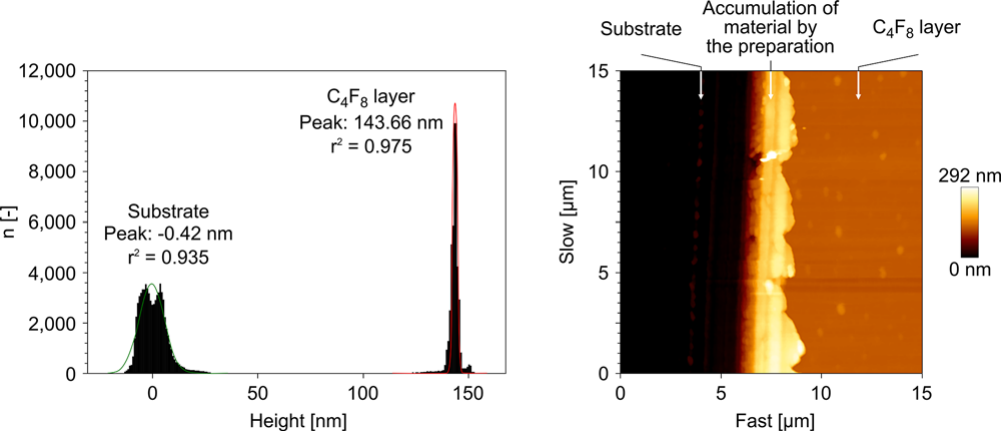
Thickness determination of the C_4_F_8_ layer by AFM. Left picture: Height difference between the substrate and the C_4_F_8_ layer. Right picture: Topography of the investigated area.

The film thickness was estimated to be in the range of ∼140 nm, since AFM measurements on the identical coatings deposited on glass revealed a film thickness of 143 nm (Fig. 2).

### 3.2 Droplet generation depending on the DMS design

#### 3.2.1 Influence on the droplet volume

**Figure 3:**
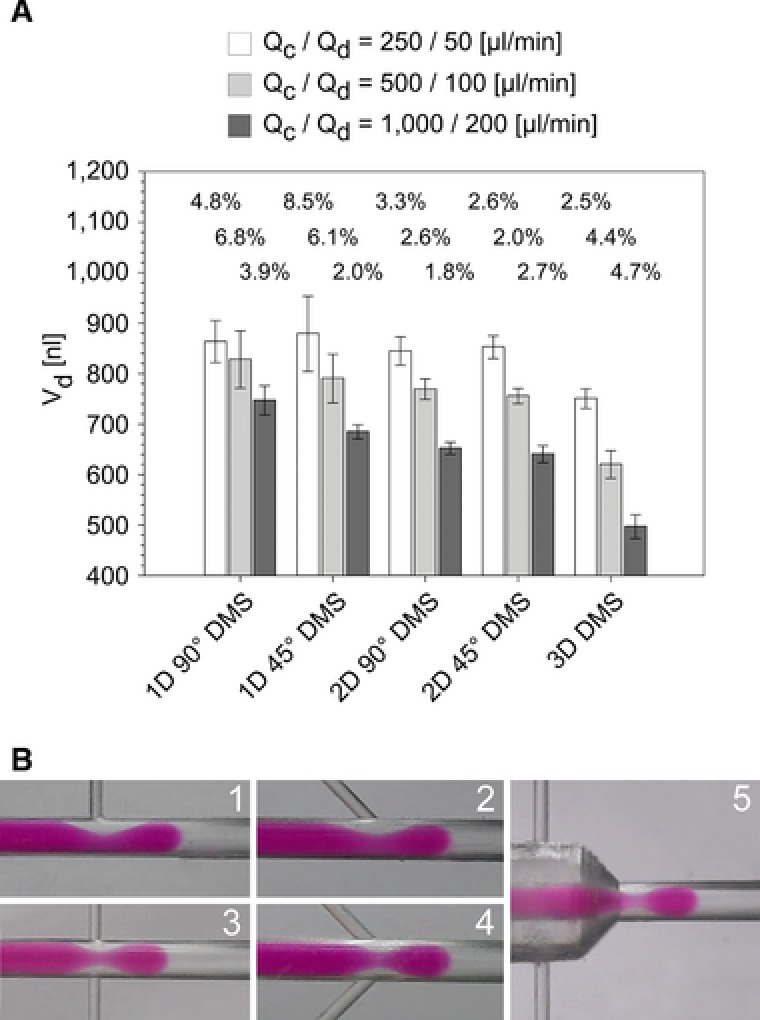
(A)‐Droplet volume with respect to the five investigated chip designs exemplarily for two flow rate ratios (data displayed as mean ± total inter‐run‐deviation for four experiments with 450 to 3500 droplets each). (B)‐Photographic images of the droplet generation of cell culture medium (DMEM, red) with PFD (colorless) for the five chip designs (Fig. 1D) at flow rates Q_c_/Q_d_ of 500/100.

The influence of the DMS design on the droplet volume is representatively presented for two flow rate combinations Q_c_/Q_d_: 500/100 and 1000/200. Being given a constant Q_c_ and Q_c_/Q_d_ ratio, the volumes of the droplets are highly dependent on the design of the DMS (Fig. 3A). It is obvious that an increasing dimensionality of the PFD injection (Table 1) caused a volume decrease of the droplets, which is most pronounced for the 3D DMS.

Although the PFD flow velocity decreased with increasing dimensionality, the injection of PFD from more than one side channel led to stronger focusing and consequently to a more intensive pinch off of the developing droplet from the aqueous sample phase, which reduced the volume of the generated droplet. Additionally, the angle at which the PFD is injected into the disperse phase also affected the droplet volume: a decreasing angle between the side channel and the main channel decreased the droplet volume (Fig. 3A). This effect could be observed for both, for one side channel as well as for two side channels. Due to the lower angle (here: 45°) the tangential force in flow‐direction was stronger and the droplet was additionally pushed to pinch off. This resulted in an accelerated break‐up of the droplet from the aqueous phase as compared to junction DMS having one or two 90° side channels. This phenomenon was more pronounced for DMS with one side channel than for DMS with two side channels.

#### 3.2.2 Influence on the droplet volume reproducibility

The influence of the DMS design on the droplet volume reproducibility was not as obvious as its influence on the droplet volume. The results of the droplet generation over multiple independent runs (total inter‐run‐deviation, Fig. 3A) show that there was a lower standard deviation for flow‐focusing DMS as compared to junction DMS except for the high flow rate runs using the 2D 45° DMS and the 3D DMS. Furthermore, the standard deviation of the droplet volume was lower for 1D 45° DMS as compared to the 1D 90° DMS. For the 2D 45° DMS and 3D DMS the opposite behavior was observed for the high flow rate runs. The PFD injection angle also influenced the droplet volume reproducibility and shall be discussed for the flow rate combination 1000/200, since there was no significant volume difference of the prepared droplets with respect to the operating time.

PFD indicates a high adherence affinity to the plasma coated channel walls (fully spread contact angle). This leads to the formation of a thin PFD film on the surface of the channel walls, which prevents the droplets from contacting the channel surface 30, 31. The presence of such a protective PFD film was demonstrated in experiments in which the fluidic system was not rinsed with PFD prior to the droplet generation process. In these experiments the sample fluid was in immediate interaction with the channel surface. This interaction was not observed when a PFD film had formed after rinsing the channels with PFD 32.

In the case of the junction DMS the injected PFD pushed the sample fluid against the PFD film being on the channel surface. The resulting pressure led to interactions between the involved interfaces. In the case of 90° DMS the PFD injection led to the largest pressure whereas in the case of 45° DMS there was a pressure component in z‐direction leading to a faster and more reproducible droplet generation (Fig. 3B). These effects were observed during the droplet generation with the 2D and 3D DMS will be discussed in the next chapter along with the influence of the flow rates.

### 3.3 Droplet generation depending on the flow rates

**Figure 4:**
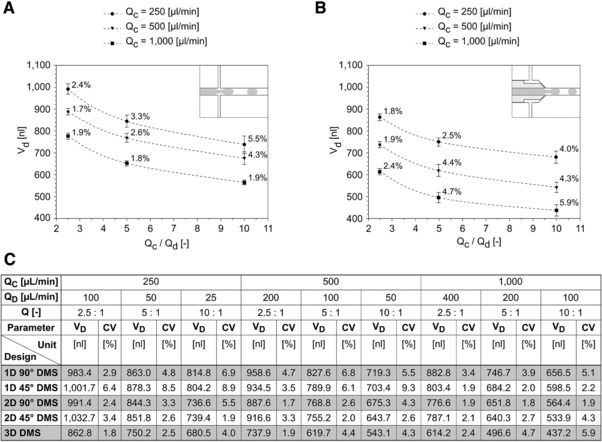
Correlation between the flow rate ratio Q_c_/Q_d_ and the resulting droplet volume for different flow rates Q_c_ for the two chip designs (A)‐2D 90° DMS and (B)‐3D DMS. Data are expressed as mean +/− standard deviation, the standard deviation is additionally expressed and depicted as CV values). (C) Droplet volume V_D_ und droplet volume deviations CV for all investigated chip designs.

The influence of the flow rates on the droplet generation process has been described in the literature 33, 34. We could show, that a wide range of droplet volumes can be chosen by adjusting the flow rates Qc and Qd and by employing different DMS designs like 2D 90  DMS (∼560 nL to ∼1010 nL) and 3D DMS (∼425 nL to ∼870 nL), respectively (Fig. 4).

#### 3.3.1 Influence on the droplet volume

Given a constant Q_d_ a proportional increase in Q_c_ resulted in a decrease of the droplet volume. Furthermore, a proportional increase of Q_d_ at a constant Q_c_ led to the formation of droplets with an increased volume. Within the investigated Q_c_/Q_d_ range the droplet volume reduction with a decreasing Q_c_ was logarithmic while there was a linear increase in the droplet volume with rising Q_d_ values. These correlations are known from the literature and could be confirmed 35, 36, 37, 38.

#### 3.3.2 Influence on the droplet volume reproducibility

A strong correlation of the reproducibility of the droplet generation process with the flow rates is displayed by the experimental results. In the case of increasing the flow rate of the disperse phase the reproducibility increased. In the case of increasing the flow rate of the continuous phase the reproducibility also increased, except for the 3D DMS, which behaved reversely. For the 2D 45° DMS there was no clear correlation regarding the reproducibility of the flow rates of both phases.

When the flow rate of the continuous phase was increased, the flow velocity rose and consequently the pressure increased. As a result the shear forces that were imposed onto the disperse phase also increased. As a consequence, there was a stronger constriction of the connection between the developing droplet and the disperse phase. The increased force of the continuous phase on the developing droplet obviously caused a faster (resulting in smaller droplet volumes) and more reproducible droplet generation.

When the flow rate of the disperse phase was increased, the flow velocity rose and consequently the injection pressure, too. This also reduced the influence of the viscosity and the interfacial tension, which significantly affected the droplet generation reproducibility. In general, increasing flow rates of the continuous or the disperse phase increased the dynamics of the droplet generation and thus the reproducibility.

Comparing the results of the 2D 90° DMS (Fig. 4A) with the 3D DMS (Fig. 4B) it is obvious that the best total inter‐run reproducibility (representing long term application) was obtained for 2D 90° DMS at high Q_c_ and for 3D DMS at low Q_c_. The reasons for this unusual behaviour of the 3D DMS shall be discussed following. During the droplet generation with the 3D DMS (notably at the flow rate Q_c_ of 1000 μL/min and the Q_c_/Q_d_ ratios of 5 and 10) it was observed, that the droplet diameter was smaller than the diameter of the microfluidic channel. Consequently, due to the missing stabilization that is normally exerted to the droplet by the wall of the microfluidic channel the droplet generation was inconstant (higher degree of freedom) causing a decreasing reproducibility.

Compared to the 90° DMS in the 3D DMS there is a distinct downstream force component caused by the flow of the continuous phase PFD. The horizontal force component pushes the developing droplet downstream. As the vertical component of the PFD flow is smaller than in the 2D 90° DMS, the squeezing zone will be influenced by the pushed developing droplet whereas the influence of the vertical PFD force component becomes smaller. Thus, the droplet pinching–off will be more influenced by stretching the squeezing zone caused by the pushed droplet than by the squeezing effect caused by the vertical component of the PFD flow.

Furthermore, in the case of 2D 90° DMS the PFD is guided from the two side channels directly into the main channel. In the case of the 3D DMS the PFD also comes from two side channels but it will be spatially distributed into an annular gap surrounding the main channel like a funnel. The abrupt junction between the side channels (arranged in 2D) and the annular gap (3D) causes undefined flow conditions leading to flow gradients with irregular flow rates. These irregular flow rates also influence the droplet generation.

### 3.4 Droplet generation depending on the sample medium

In addition to the DMEM medium further fluidic samples (Table 3) were subjected to droplet generation, as well. Droplets were generated from these media employing the 2D 90° DMS for 8 h. The quality of the droplet generation process (and thus the stability of the coating) was assessed qualitatively in a range from droplet generation without any signs of problems (“+”), slight adhesion of the droplet with the channel wall (“0”) and heavily disturbed droplet generation with significant droplet adhesion (“‐”).

Almost all of the investigated application relevant media could be segmented over a period of 8 h with a high quality, indicating that the DMS coating was stable over that period. However, whole blood (porcine, anticoagulated with citrate) and human plasma slightly reduced the quality of the droplet generation process. For whole blood a perfect droplet generation was observed initially. After a few minutes the whole blood non‐cyclically interacted with the channel wall downstream the droplet generation zone. This adhesion of droplets was predominantly observed at sites at which the lowest thickness of the PFD film was expected (compare chapter 3.2). At the sites of the reduced PFD film thickness the probability of an adhesion of medium components to the channel surface obviously increases. The adhesion induced changes of the surface properties were irreversible and resulted in a decreased reproducibility of the droplet generation process.

Varying the flow rates affected the droplet adhesion significantly. At flow rates of 250/25 the adhesion tendency increased while it was almost completely inhibited when the flow rates were raised to 500/100. This may be attributed to the short time period in which an individual droplet may interact with the surface.

The exact reason for the high adhesion potential of blood to the channel walls could not be fully elucidated, yet. Since similar effects were observed for both anticoagulated whole blood and plasma (whole blood without cells) it is highly unlikely, that the blood cells play a major role in the changes at the interface (although the negative effects were more pronounced for whole blood). Consequently, it is more likely that coagulation factors interfere with the channel surface, which mask the surface and thus change its physico‐chemical properties. The adhesion would likely lead to an initiation of the coagulation cascade, which was suppressed by the added citrate in these experiments (no signs of fibrin polymerization were observed, surface confined effect). The hypothesis that coagulation factors may interfere with the droplet generation is further supported by the observation, that media (FBS, DMEM and IMDM) containing serum (i.e., plasma depleted from coagulation factors) supported a high quality droplet generation process. However, further research on the adhesion of blood cells and coagulation factors will be performed in order to support this hypothesis.

Even more dramatic, negative effects on the droplet generation process were observed when media with extreme pH values were employed. After the generation of a few initial droplets, media with a pH 10 and pH 2 could not be segmented in the DMS but only at the transition zone from the DMS to the PTFE tubing. Since this is a phenomenon usually only observed for uncoated DMS we hypothesize that the coating is not stable against high concentrations of H^+^ and OH^−^ ions. We also suspect that the coating is irreversibly damaged since even after rinsing the system with deionized water and PFD the droplet generation process of DMEM was severely affected. The detailed elucidation of how the high H^+^ and OH^−^ concentrations interfere with the coating (detachment or modification) is the subject of ongoing research.

## 4 Concluding remarks

A plasma coating of PC materials with the c‐C_4_F_8_ precursor is a perfectly suited technology for equipping the surface of microfluidic systems with the hydrophobicity that supports a stable droplet generation process. Consequently, it is possible to perform the droplet generation process without surfactants, which may negatively interfere with the bio(techno)logical applications. By choosing an appropriate DMS design and adjusting the flow rates, the droplet volumes could be tuned in a range of ∼435 nL to ∼1065 nL.

One of the most relevant parameters of the DMS was its reproducibility, which was dependent on the DMS design and the flow rates. Independent of the design all DMS revealed an excellent reproducibility for short term applications. Furthermore, a high reproducibility for long term applications was observed for high flow rates. These high flow rates of 1000 μL/min resulted in a higher dynamic of the droplet generation with a low variation in droplet volume and are thus preferable. A higher dimensionality of the PFD injection led to an improved reproducibility of the droplet generation process. Similarly, the 2D injection at an angle of 90° resulted in a superior reproducibility as compared to an injection at 45°. Consequently, the 2D 90°DMS is a promising DMS design for a reproducible droplet generation process. Further research will be focused on the development and characterization of the 3D DMS since it is highly likely, that this combination of the dimensionality and the 90° injection angle may further improve the reproducibility.

The upper limit for the Q_c_ flow rate was 2000 μL/min which corresponds at a Q_c_/Q_d_ ratio of 5 to a droplet generation frequency of 10 s^−1^. Further increasing of the Q_c_ flow rate caused decreasing distances between the droplets which led to undesired droplet contact and, as we operate without surfactants, to fusion of droplets.

The here presented plasma coated DMS were demonstrated to be well applicable for the reproducible generation of droplets from a wide variety of bio(techno)logically relevant media. Even the droplet generation from anticoagulated blood products (like whole blood and plasma) was possible although it was less reproducible due to an increased adhesion of droplets to the channel walls in 2D DMS. However, using the 3D DMS this adhesion should be significantly reduced because of the much more pronounced PFD film between the droplets and the channel surface caused by the 3D flow‐focusing effect. Adhesion phenomena depending on the chip geometry and the used fluids will be investigated further.

The results presented in this study are the basis for further development of functional chips and applications with delicate samples like suspensions of (multi)cellular systems or even tissue fragments. Both, polymer as DMS material and DMS preparation by plasma coating are convenient and cost effective. Consequently, it will be possible to offer the systems as single use devices for high‐throughput applications in industry and academia.

**Practical application**

Droplet based microfluidics has a promising application potential in the field of drug screening for personalized medicine or for pharmaceutical research, to mention only a few. Precondition for all applications is a stable and reproducible droplet generation and manipulation as well as cost‐effective equipment like microfluidic chips. In this study we present microfluidic polymer chips that are capable to generate and manipulate aqueous droplets highly reproducibly and without the use of surfactants. The droplets could be used to cultivate even mammalian cells e.g. as spheroids or tissue fragments. The chips could be manufactured by both precision manufacturing, e.g. milling, and injection molding for use as disposables. Thus, for each application the microfluidic channel structure could be milled and tested before molding a higher number of disposable chips. Each chip consists of two symmetrical chip‐halves that can be coated application‐related before assembly.

*The authors have declared no conflict of interest*.

## Nomenclature

**Table:** 

d_c_	[μm]	Diameter of the main channel of the PC chips
d_OD1_	[μm]	Diameter of the first optical detector
d_OD2_	[μm]	Diameter of the second optical detector
*n*	[‐]	Droplet index
*n_max_*	[‐]	Number of all generated droplets in a run
Q_c_	[μL/min]	Volumetric flow rate of the continuous phase (organic phase, separation medium, perfluorodecaline)
Q_d_	[μL/min]	Volumetric flow rate of the disperse phase (aqueous or sample phase, biological media)
S_a_	[μm]	Arithmetical mean height of the surface (area roughness)
s_D,nOD_	[μm]	Droplet length calculated from the optical detector singals
s_D,OD_	[μm]	Average droplet length calculated from the optical detector signals
s_D,SP_	[μm]	Average droplet length calculated from the syringe pump level
s_TD,nOD_	[μm]	Transformed droplet length calculated from the optical detector signals
*T_s/s_*	[‐]	Transformation factor
